# Role of stemness‐related genes TIMP1, PGF, and SNAI1 in the prognosis of colorectal cancer through single‐cell RNA‐seq

**DOI:** 10.1002/cam4.5833

**Published:** 2023-04-05

**Authors:** Yan Shen, Siyi Ni, Si Li, Bin Lv

**Affiliations:** ^1^ Department of Gastroenterology The Second Affiliated Hospital of Zhejiang Chinese Medical University Hangzhou Zhejiang China; ^2^ Department of Pediatrics Tongde Hospital of Zhejiang Province Hangzhou Zhejiang China; ^3^ Department of Gastroenterology The First Affiliated Hospital of Zhejiang Chinese Medical University Hangzhou Zhejiang China

**Keywords:** colorectal cancer, in vitro cell experiment, pseudotime trajectories analysis, single‐cell RNA‐seq, stemness‐related prognostic genes

## Abstract

**Background:**

Colorectal cancer (CRC) is a fatal malignant tumor with poor prognosis. Cancer stem cells (CSCs) can cause metastasis, recurrence and drug resistance in CRC. This research aimed to analyze stemness‐related prognostic genes of CRC based on single‐cell RNA‐sequencing (scRNA‐seq) data.

**Methods:**

DESeq2 was applied to analyze the differentially expressed genes (DEGs). The mRNA stemness index (mRNAsi) was calculated by one‐class logistic regression (OCLR). The stemness‐related cells were analyzed based on scRNA‐seq dataset GSE166555. Monocle 2 algorithm was used for stemness‐related cells pseudotime trajectory analysis. The stemness‐related prognostic genes were analyzed by clusterProfiler and survival package. The stemness of CRC cells was detected by spheroid formation assay, and the expression of stemness‐related prognostic genes was verified by qRT‐PCR and Western blot.

**Results:**

7916 DEGs between the CRC and normal tissues were obtained. The mRNAsi of the CRC tissues was shown to be significantly higher than that of the normal tissues. 7 and 8 cell types were annotated respectively in the normal and CRC tissues through analysis of the scRNA‐seq data. Cell–cell interactions (CCIs) in the tumor tissues were revealed to be significantly enhanced than that in the normal tissues. By calculating the ‘stemness score’, CSCs, epithelial cells (EPCs) and cancer‐associated fibroblasts (CAFs) were defined as stemness‐related cells. Through pseudotime trajectory analysis, 2111 genes were identified as state 2‐specific genes. Then, 41 genes were obtained by taking intersection of the up‐regulated genes with state 2‐specific genes and marker genes of CSCs, EPCs and CAFs. The univariate COX regression analysis revealed 5 stemness‐related prognostic genes (TIMP1, PGF, FSTL3, SNAI1 and FOXC1). Kaplan–Meier curve analysis indicated that the higher the expression of 5 genes, the lower the survival rate. In vitro cell experiment confirmed that the expression of TIMP1, PGF and SNAI1 was consistent with that revealed by bioinformatics analysis.

**Conclusions:**

TIMP1, PGF and SNAI1 were identified as stemness‐related prognostic genes of CRC, and possibly potential therapeutic targets for CRC.

## Graphical abstract

Flow chart for screening stemness‐related prognostic genes of CRC.

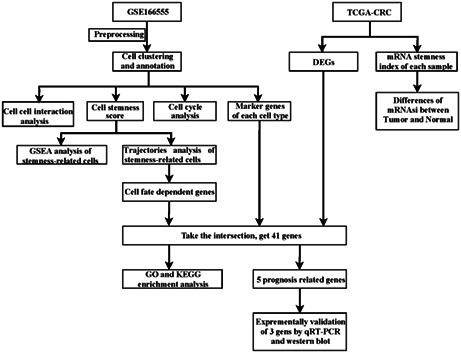

## 1 INTRODUCTION

According to the global cancer statistics in 2020, colorectal cancer (CRC) is a severe and fatal malignant tumor with the third highest incidence and the second highest mortality worldwide.^1^ Although treatment strategies such as surgery, chemotherapy, radiotherapy, immunotherapy, and targeted therapy have certain inhibitory effects on the development of CRC, the survival rate of CRC patients is still very low,^2^ and varies greatly for patients at different pathological stages. Therefore, there are still great challenges for the prognosis of CRC.^3^ In addition, the heterogeneity of CRC makes the decisions on its treatment difficult.^4^, ^5^ Although many biomarkers associated with the prognosis of CRC have been previously reported,^6^ it is still of great significance to find new prognostic biomarkers of CRC as its potential therapeutic targets. Therefore, bioinformatics analysis was performed in this study to search for new prognostic genes of CRC based on single‐cell RNA‐sequencing (scRNA‐seq) data.

Cancer stem cells (CSCs), as a special cell type in the cancer cell population, have self‐renewal capacity, and are involved in the occurrence, metastasis, recurrence, and drug resistance of tumors, thus affecting tumor progression and prognosis.^6^, ^7^ Colon cancer stem cells (CCSCs) have been reported to be closely related to the development and prognosis of CRC, and LGR5 has been found to be a marker of CSCs for the poor prognosis of CRC.^8^ One‐class logistic regression (OCLR) is a machine learning algorithm applied in this study to determine the mRNA expression of pluripotent stem cells and their differentiated progenies for calculating the single‐cell mRNA expression stemness index (mRNAsi), with an aim to reveal the tumor stemness‐related heterogeneity.^9^ Ye et al^10^ determined the mRNAsi scores in CRC and introduced a stemness‐related classification to predict patient outcomes based on The Cancer Genome Atlas (TCGA) database. On the basis of existing researches, this study is expected to find new stemness‐related prognostic genes of CRC.

In order to identify prognostic genes related to CRC stemness, we comprehensively analyzed the CRC data from TCGA database and the scRNA‐seq data from the Gene Expression Omnibus (GEO) database. In addition, the expression of stemness‐related prognostic genes was validated by CRC in vitro cell experiment. These stemness‐related prognostic genes were determined as potential therapeutic targets for CRC.

## 2 MATERIALS AND METHODS

### 2.1 Data acquisition

The mRNA‐sequencing (mRNA‐seq) and clinical data of CRC were obtained from TCGA (TCGA‐CRC includes TCGA‐COAD and TCGA‐READ). After collation, 653 samples with corresponding survival data were retained, including the sample data of 605 CRC tumor tissues and 48 adjacent tissues. The clinical characteristics of the samples were listed in Table 1. The CRC scRNA‐seq data (GSE166555) was downloaded from the GEO, which contained corresponding data of 13 tumor tissues and 12 para‐carcinoma tissues. The scRNA‐seq defined the cell composition of normal and tumor intestinal tissues.

**TABLE 1:** The clinical characteristics of samples from TCGA.

Variables	Number (*N* = 605)
OS	
Live	481
Death	124
Age	
≤65	267
>65	338
Gender	
Male	328
Female	277
Stage	
Stage I	106
Stage II	219
Stage III	174
Stage IV	85
Unknown	21
T	
T1	20
T2	106
T3	411
T4	65
Unknown	3
M	
M0	449
M1	84
Unknown	72
N	
N0	344
N1	146
N2	111
Unknown	4

### 2.2 Differentially expressed genes (DEGs) and mRNAsi analysis of TCGA‐CRC

The DEGs between tumor and normal tissues were analyzed by DESeq2 (|logFC| > 1, *p*adj < 0.05), and a volcano plot was drawn. OCLR was applied to calculate the mRNAsi of tumor and normal tissues.^9^ The difference of mRNAsi between tumor tissues and adjacent tissues was detected by *t* test.

### 2.3 Analysis of the scRNA‐seq data in GSE166555

The count data was obtained from GSE166555 and subjected to quality control. The first 2000 genes with the most significant difference between cells were identified by FindVariableFeatures. Thirteen tumor tissues and 12 para‐carcinoma tissues were integrated respectively by ‘IntegrateData’ for batch effect elimination. Principal component analysis (PCA) was applied to identify available dimensions (*p* < 0.05). Then, the algorithm of t‐SNE was used to reduce the dimensions of 20 initial principal components (PCs), and to perform cluster classification analysis across all cells. The cell types of the clusters were annotated based on the cell markers in Table S1.^11^, ^12^

### 2.4 Analysis of cell–cell interactions (CCIs)

To identify the differences of CCIs between normal and tumor tissues, the CCI score representing the communication probability among all subclusters based on ligand‐receptor pairs was calculated,^13^ which summarized the scores of all pathways between two kinds of cells. A network diagram was built, and a thicker edge indicated a higher degree of cell–cell interaction.

### 2.5 Analysis of stemness‐related cells

We defined the ‘stemness score’ to facilitate the quantitative assessment of stemness in each cell type. In all single cells, the average relative expression of stemness‐related genes was calculated.^14^, ^15^ Then, the cell cycle of each cell type was estimated using the seurat CellCycleScoring function. Lastly, Gene Set Enrichment Analysis (GSEA) was used to complete Gene Ontology (GO) functional enrichment analysis with molecular marker technology database (MSigDB) C5GO: BP (Version 7.0) on cells with high stemness scores. The ‘FindAllMarkers wilcox’ was applied to identify markers in each cell type (min.pct = 0.2, logfc.threshold = 0.25).

### 2.6 Stemness‐related pseudotime trajectory analysis

The single‐cell pseudotime trajectory of CSCs, epithelial cells (EPCs), and cancer‐associated fibroblasts (CAFs) was established using the algorithm of Monocle 2. Single cells were projected onto a space, forming a trajectory with branch points. It is generally considered that cells in an identical branch are in the same state of differentiation. Moreover, the branch point was analyzed using the method of branched expression analysis modeling (BEAM) (qval < 1e‐20). Genes in the branch point were clustered, and branch‐dependent or state‐specific genes were identified.^16^

### 2.7 Identification of stemness‐related prognostic genes

The up‐regulated genes in TCGA‐CRC were intersected with the state‐specific genes from trajectory analysis, and the marker genes of CSCs, EPCs, as well as CAFs first. Second, clusterProfiler was applied for GO and Kyoto Encyclopedia of Genes and Genomes (KEGG) enrichment analysis of the common genes. Thirdly, univariate COX regression analysis was performed on the common genes using the survival package to identify prognostic‐related genes (threshold *p* < 0.05, hazard ratio (HR) > 1).^17^

### 2.8 Analysis of stemness‐related prognostic genes

The expression of stemness‐related prognostic genes in the eight cell types was estimated, and a violin plot was drawn using Seurat. Moreover, Kaplan–Meier curve analysis was performed.^18^

### 2.9 Cell culture

CRC cell lines (HCT116 and DLD1) and normal colonic epithelial cells (NCM460) were derived from the Cell Bank of China Academy of Sciences. HCT116 and DLD1 were cultivated in RPMI 1640 (Gibco Life Technologies), and NCM460 was cultivated in DMEM (Gibco Life Technologies) at 37°C with 5% CO_2_.

### 2.10 Spheroid formation assay

HCT116 and DLD1 (1 × 10^4^cells/mL) were placed in the six‐well ultra‐low attachment culture plate and cultured in serum‐free DMEM/F12 (Gibco Life Technologies) medium for 1 week. Then, the image of cell formation was observed under a microscope.

### 2.11 qRT‐PCR assay

The total RNA was collected from HCT116, DLD1, and NCM460 using the Trizol kit (Invitrogen), and cDNA was synthesized. qRT‐PCR was performed with β‐actin as an internal reference. The gene expression levels were computed by 2^−ΔΔCT^ three times repeatedly (Table 2).

**TABLE 2:** The primers used in qRT‐PCR assay.

Gene	Sequence (5′–3′)
TIMP1 Forward primer	GACGGCCTTCTGCAATTCC
TIMP1 Reverse primer	GTATAAGGTGGTCTGGTTGACTTCTG
PGF Forward primer	ACTTGGGAACACAAGAAGCCT
PGF Reverse primer	CGACCCCACACTTCGTTGAA
FSTL3 Forward primer	ACGTTACCTACATCTCGTCGTGTC
FSTL3 Reverse primer	GGTTTCATGGTCGTCTCCTCCT
SNAI1 Forward primer	TTTACCTTCCAGCAGCCCTA
SNAI1 Reverse primer	GACAGAGTCCCAGATGAGCA
FOXC1 Forward primer	AGAAGGACAGGCTGCACCTCA
FOXC1 Reverse primer	GTTCTCGGTCTTGATGTCCTGG
β‐actin Forward primer	GCACCACACCTTCTACAATGAGC
β‐actin Reverse primer	GGATAGCACAGCCTGGATAGCAAC

### 2.12 Western blot assay

The total protein was collected from HCT116, DLD1, and NCM460 using RIPA lysis buffer, and the protein concentration was measured using the BCA kit (Beyotime Biotechnology Company). After separation by gel electrophoresis, the proteins were transferred to the PVDF membrane and sealed with 5% skimmed milk for 1 h. Then, the proteins were reacted overnight with the primary antibodies (TIMP1, #MA5‐13688, 1:1000; PGF, #MA5‐32855, 1:1000; FSTL3, #PA5‐79285, 1:1000; SNAI1, #MA5‐14801, 1:1000; FOXC1, #PA1‐807, 1:1000; Invitrogen) at 4°C, followed by reaction with the secondary antibody (IgG/HRP, 1:2000, Thermo Fisher Scientific) for 1 h. At last, the visual protein bands were detected with ECL reagent.

### 2.13 Statistical analysis

Bioinformatics analysis was performed using the R package, and all the experimental data were analyzed using the software of GraphPad Prism 8.0. *p* < 0.05 was considered statistically significant. The difference between groups was detected by *t* test. DEGs were analyzed using DESeq2 (|logFC| > 1, *p*adj < 0.05), and the univariate COX regression analysis was performed using the survival package to identify prognostic‐related genes (threshold *p* < 0.05, hazard ratio (HR) > 1).

## 3 RESULTS

### 3.1 Analysis of the DEGs and mRNAsi in TCGA‐CRC

**FIGURE 1:**
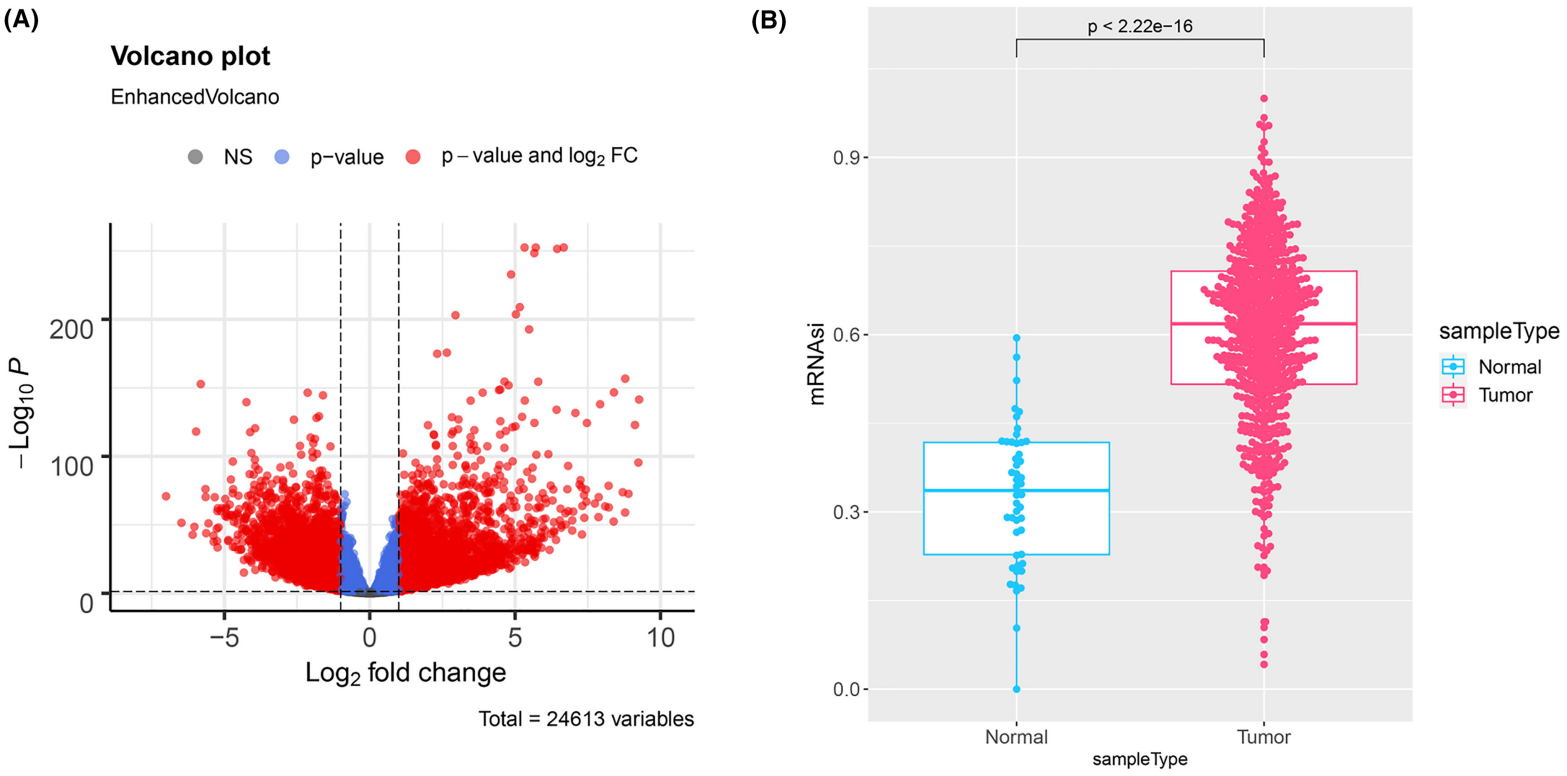
Analysis of the differentially expressed genes (DEGs) and mRNAsi in TCGA‐CRC. (A) The volcano plot of DEGs. (B) The mRNAsi of tumor and normal tissues.

7916 DEGs were analyzed using DESeq2, including 4918 up‐regulated genes and 2998 down‐regulated genes between the CRC tumor tissues and adjacent tissues (Figure 1A). The mRNAsi of each tissue sample was calculated by OCLR, and the high mRNAsi score may be a risk factor for the overall survival of patients with tumors.^19^ Results showed that the mRNAsi of tumor tissues was significantly higher than that of the normal tissues (Figure 1B). These findings indicated that the stemness of CRC tumor tissues was significantly higher than that of the normal tissues.

### 3.2 Identification of cell clusters in scRNA‐seq dataset GSE166555

**FIGURE 2:**
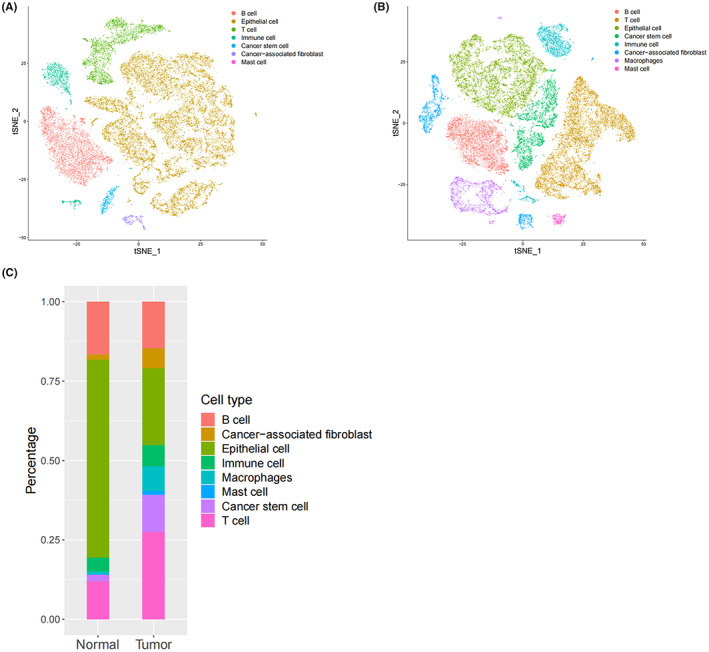
Cell types in tumor tissues (tumor) and para‐carcinoma tissues (normal). (A) The cell types in para‐carcinoma tissues. (B) The cell types in tumor tissues. (C) The cell distribution of tumor tissues and para‐carcinoma tissues.

Thirteeen tumor matrices and 12 normal matrices were integrated with the ‘IntegrateData’ function respectively. Then, 25,903 cells and 21,518 genes were contained in the normal tissue object, and 34,111 cells and 23,051 genes were contained in the tumor tissue object. The normal tissues were clustered into 19 clusters, while the tumor tissues were clustered into 16 clusters through PCA and t‐SNE (Figure S1). The cell types of cell clusters were annotated based on the cell markers in Table S1, and there were B cells, T cells, EPCs, CSCs, immune cells, CAFs, macrophages, and mast cells (Figure 2A,B). Moreover, great difference was shown in the cell distribution between tumor tissues and para‐carcinoma tissues (Figure 2C).

### 3.3 Analysis of CCIs in both tumor tissues and para‐carcinoma tissues

**FIGURE 3:**
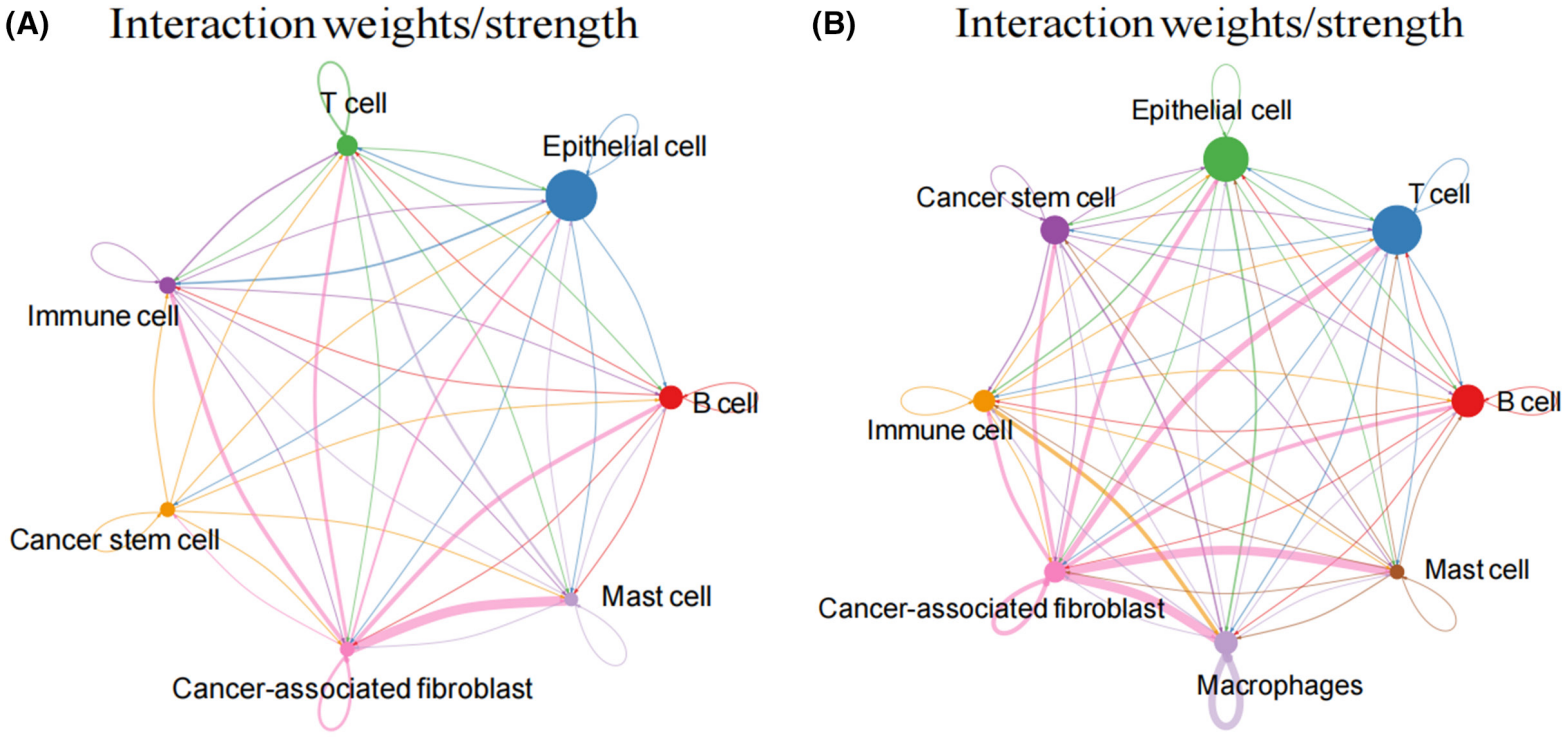
Analysis of the cell‐cell interactions (CCIs) in both tumor tissues and para‐carcinoma tissues. (A) CCIs in the para‐carcinoma tissues. (B) CCIs in the tumor tissues. A thicker edge between cells indicated a higher degree of interaction.

The results of CCIs indicated that the interactions between cells (B cells, T cells, EPCs, CSCs, immune cells, CAFs, macrophages, and mast cells) in the tumor tissues were significantly increased and stronger than that in the normal tissues, and the degree of CCIs between CAFs and other cells were particularly higher (Figure 3A,B).

### 3.4 Identification of stemness‐related cells based on the stemness score and cell cycle analysis

**FIGURE 4:**
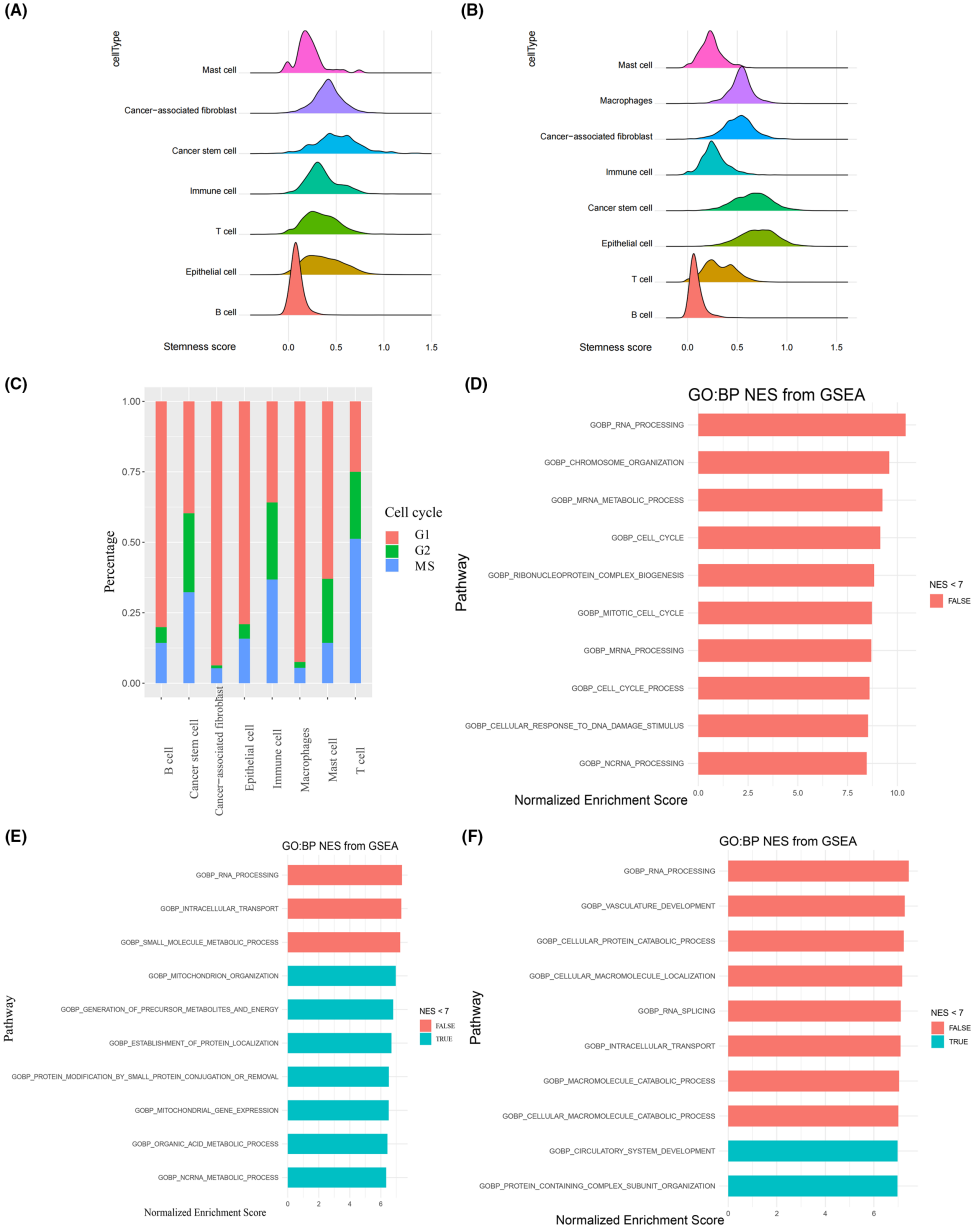
Identification of stemness‐related cells and GSEA. (A) The stemness scores of the 7 cell types in the para‐carcinoma tissues. (B) The stemness scores of the 8 cell types in the tumor tissues. (C) Cell cycle analysis of the 8 cell types in the tumor tissues. (D) GSEA of CSCs. (E) GSEA of EPCs. (F) GSEA of CAFs. True represents normalized enrichment score (NES) <7; False represents NES > 7.

We calculated the ‘stemness score’ of each cell, and results revealed that CSCs had the highest stemness score in both the tumor tissues and para‐carcinoma tissues (Figure 4A,B). Furthermore, in the tumor tissues, EPCs had the similar stemness score as CSCs, and CAFs also had relatively high stemness score, while fully differentiated B cells had the lowest stemness score (Figure 4B). Then, it was further revealed by cell cycle analysis in the tumor tissues that the cell cycle of CSCs was almost evenly distributed in the G1/S/G2/M phase, while most EPCs and CAFs were in the G1 phase (Figure 4C). To some extent, CSCs, EPCs, and CAFs were considered as stemness‐related cells. Lastly, through GSEA of CSCs, EPCs, and CAFs, CSCs were mainly enriched in RNA processing, chromosome organization, cell cycle, etc. (Figure 4D); EPCs were mainly enriched in RNA processing, intracellular transport, etc. (Figure 4E); CAFs were mainly enriched in RNA processing, cellular protein catabolic process, intracellular transport, etc. (Figure 4F). These results indicated that stemness‐related cells (CSCs, EPCs, and CAFs) were related to the development of CRC.

### 3.5 Pseudotime trajectory analysis of CSCs, EPCs, and CAFs

**FIGURE 5:**
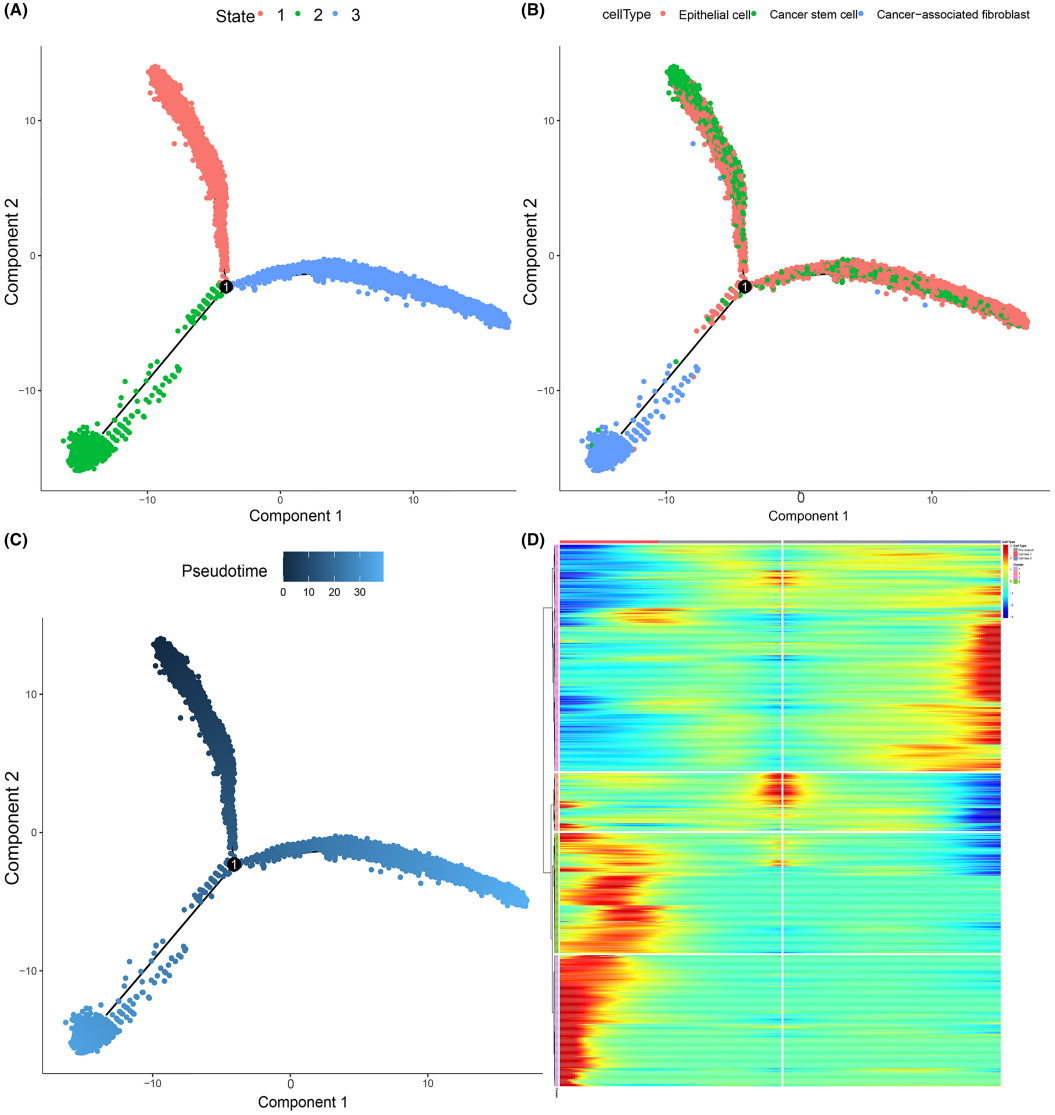
Pseudotime trajectory analysis of CSCs, EPCs, and CAFs. (A) Trajectory plot by three states. (B) Trajectory plot by three cell types. (C) Trajectory plot by pesudotime. (D) The expression heat map of branch point 1‐dependent genes; pre‐branch represents state 1, cell fate 1 represents state 2, and cell fate 2 represents state 3; blue represents low‐expression, and red represents high‐expression.

We performed pseudotime trajectory analysis on CSCs, EPCs, and CAFs. The arrangement of cells on the pseudotime line was CSCs and EPCs to CAFs, and cells in state 2 were mainly CAFs (Figure 5A–C). Next, branch point 1 was analyzed based on the method of BEAM, and 4501 genes were clustered (qval < 1e‐20). These genes were clustered into four classes (cluster 1–4, Figure 5D), and there were 2111 genes in cluster 1 and cluster 4 which were highly expressed in cell fate 1 (state 2). These genes were defined as state 2‐specific genes.

### 3.6 Identification of stemness‐related prognostic genes

**FIGURE 6:**
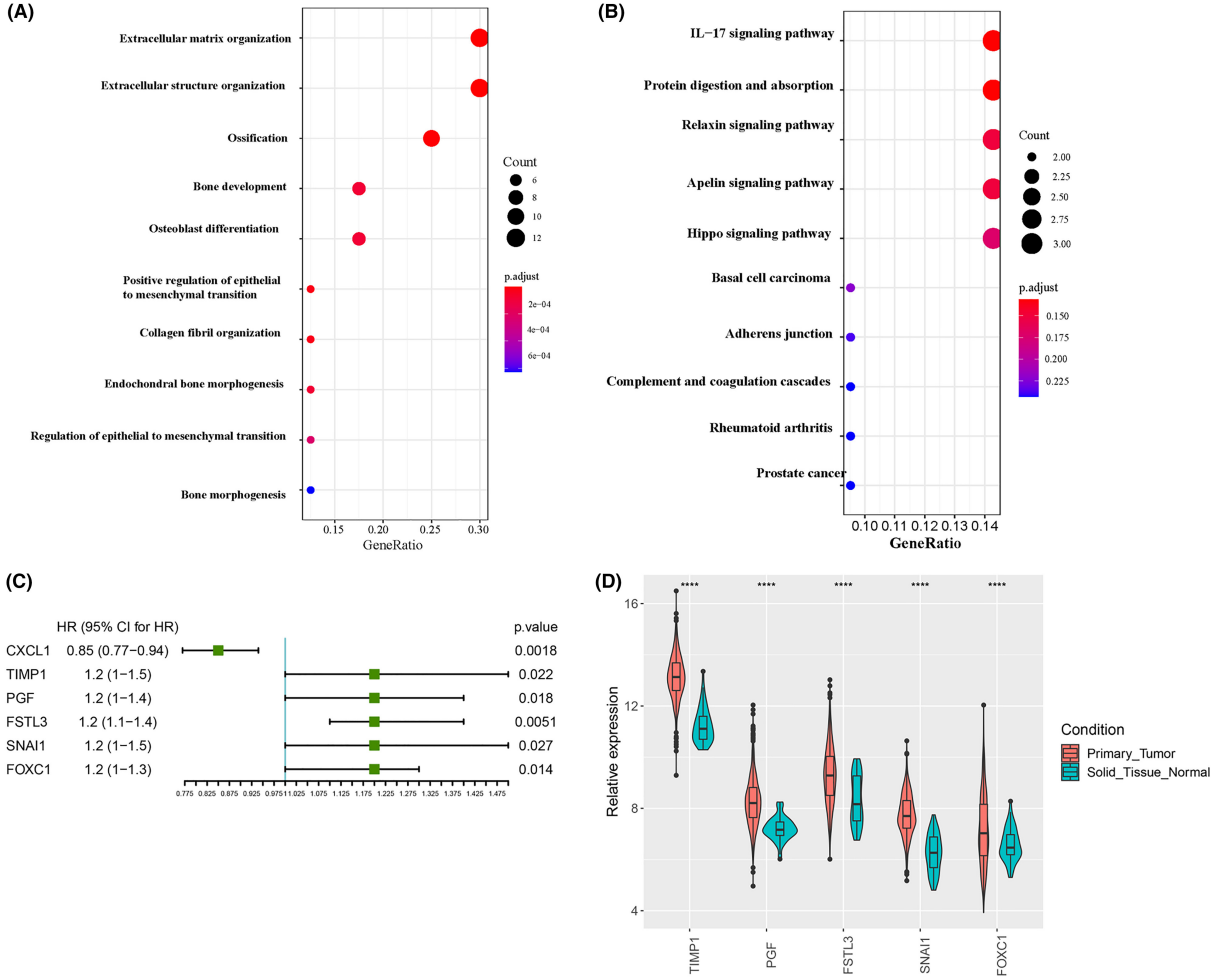
Identification of stemness‐related prognostic genes. (A) GO enrichment analysis of the 41 common genes. (B) KEGG enrichment analysis of the 41 common genes. (C) Forest plot of the 6 prognosis‐related genes. (D) Expression of the five stemness‐related prognostic genes in the normal and tumor tissues.

The marker genes of cell clusters screened by ‘FindAllMarkers wilcox’ were up‐regulated (Table S2). Then, 4918 up‐regulated genes in TCGA‐CRC were intersected with the state 2‐specific genes through BEAM analysis and marker genes of CSCs, EPCs, and CAFs. As a result, 41 genes were obtained. GO and KEGG analysis indicated that these genes were mainly enriched in the positive regulation of epithelial‐mesenchymal transition, IL‐17 signaling pathway, and hippo signaling pathway (Figure 6A,B). To identify the prognostic genes, univariate COX regression analysis was performed on the 41 genes (*p* < 0.05). There were six prognostic genes (CXCL1, TIMP1, PGF, FSTL3, SNAI1, and FOXC1), among which five stemness‐related prognostic genes (HR > 1) were obtained (Figure 6C). Moreover, the expressions of TIMP1, PGF, FSTL3, SNAI1, and FOXC1 were significantly up‐regulated in the tumor tissues (Figure 6D).

### 3.7 Expression and Kaplan–Meier curve analysis of stemness‐related prognostic genes

**FIGURE 7:**
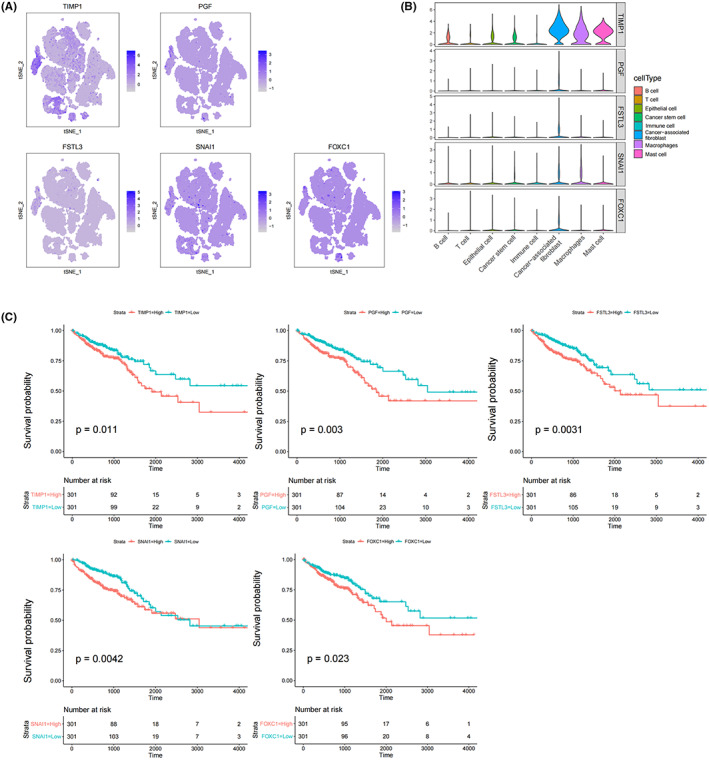
Expression and Kaplan–Meier curve analysis of stemness‐related prognostic genes. (A) Expression of the five genes in eight cell types (the darker the color, the higher the expression). (B) Violin plot of the five genes (The height denotes the gene expression level, and the width denotes the number of cells expressing five genes.). (C) Kaplan–Meier curve of the five genes

TIMP1, PGF, FSTL3, SNAI1, and FOXC1 were highly expressed in CAFs with high stemness scores in the tumor intestinal tissues (Figure 7A,B). Then, the results of Kaplan–Meier curve analysis indicated that the higher the expression of the stemness‐related prognostic genes, the lower the survival rate (Figure 7C). Based on the above results, we speculated that the high expression of stemness‐related prognostic genes (TIMP1, PGF, FSTL3, SNAI1, and FOXC1) were related to the development of CRC, and affected the survival rate of patients with CRC.

### 3.8 Expression verification of stemness‐related prognostic genes in vitro

**FIGURE 8:**
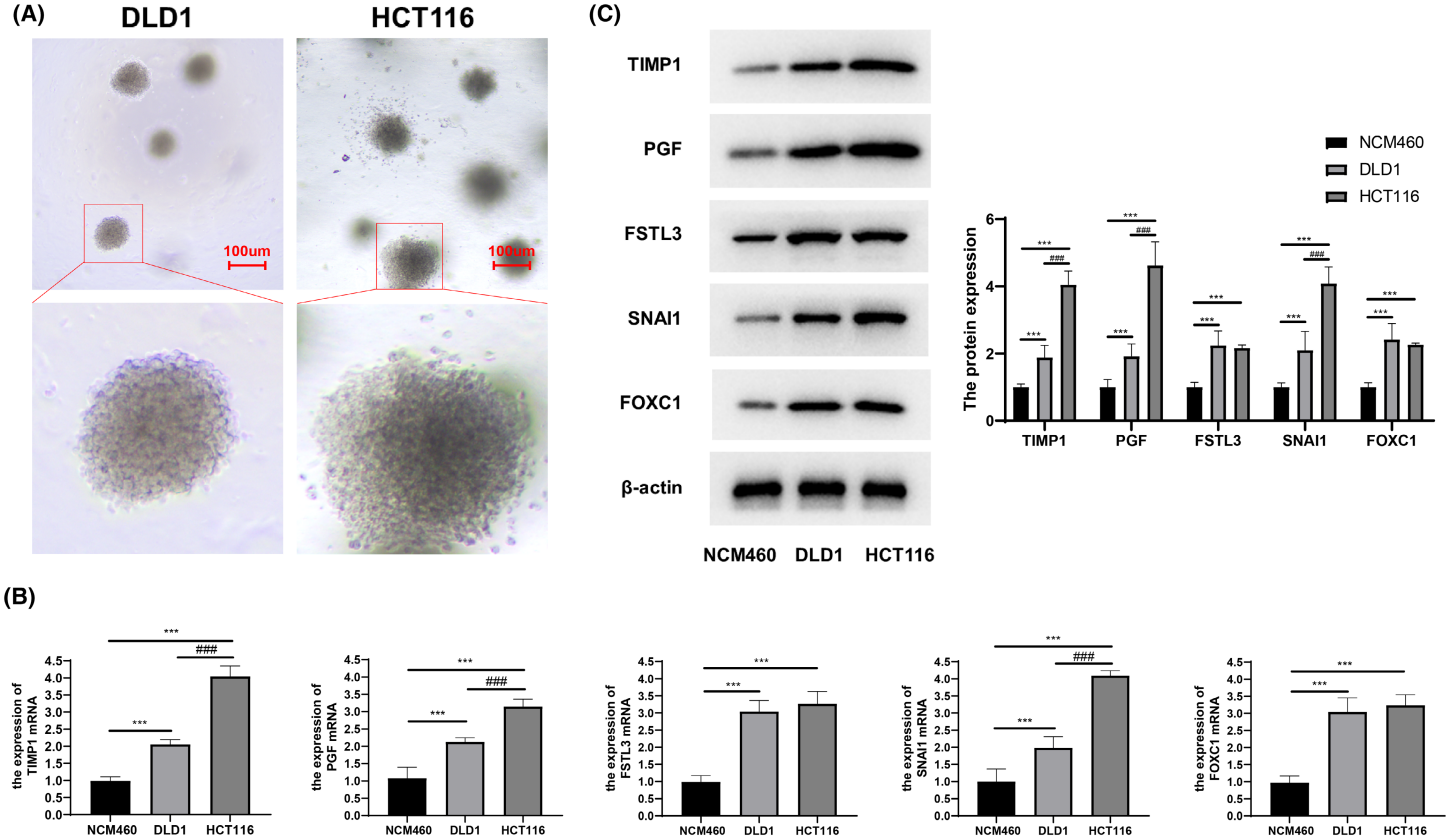
Expression verification of stemness‐related prognostic genes. (A) The spheroid formation assay of HCT116 and DLD1. (B) The expressions of TIMP1, PGF, FSTL3, SNAI1, and FOXC1 in NCM460, HCT116, and DLD1 detected by qRT‐PCR. (C) The expressions of TIMP1, PGF, FSTL3, SNAI1, and FOXC1 in NCM460, HCT116, and DLD1 detected by western blot; compared with NCM460, ***p < 0.001; compared with DLD1, ^###^ *p* < 0.001.

In the CRC subtyping system, HCT116 belongs to stem‐cell phenotype, while DLD1 belongs to other phenotypes.^20^, ^21^ Spheroid formation assay revealed that the sphere formation ability of HCT116 was significantly higher than that of DLD1, indicating that the stemness of HCT116 was stronger than that of DLD1 (Figure 8A). The gene expression level was detected by western blot and qRT‐PCR (Figure 8B,C). Compared with NCM460, TIMP1, PGF, FSTL3, SNAI1, and FOXC1 were significantly up‐regulated in CRC cells (HCT116 and DLD1). Moreover, the expression of TIMP1, PGF, and SNAI1 was relatively higher in HCT116 with stronger cell stemness. FSTL3 and FOXC1 were slightly up‐regulated, but there was no significant difference between HCT116 and DLD1.

## 4 DISCUSSION

Poor prognosis and low survival rate are two thorny problems in the clinical treatment of CRC.^22^ Screening of biomarkers related to the overall survival of CRC patients is of great significance, because it is helpful for selecting appropriate CRC treatment regimens and improving the prognosis of CRC patients.^23^ In this research, with the aid of DESeq2, we analyzed the DEGs between CRC and normal tissues based on the TCGA‐CRC data (TCGA‐COAD and TCGA‐READ), and there were 7916 DEGs, including 4918 up‐regulated genes and 2998 down‐regulated genes. Moreover, the mRNAsi of the tumor tissues was significantly higher than that of the normal tissues, indicating that the stemness of CRC tumor tissues is stronger than that of the normal tissues.

The scRNA‐seq data in GSE166555 were analyzed. Eight cell types (B cells, T cells, EPCs, CSCs, immune cells, CAFs, macrophages, and mast cells) were annotated in cell clusters, and there was a great difference in the cell distribution between tumor tissues and para‐carcinoma tissues. The analysis results of CCIs indicated that the interactions between cells in the tumor tissues were significantly increased and stronger than that in the normal tissues, and the degree of CCIs between CAFs and other cells were particularly higher. To some extent, it can be considered that CAFs were important in the progression of CRC. Then, CSCs, EPCs, and CAFs were defined as stemness‐related cells based on the stemness score and cell cycle analysis. GSEA showed that CSCs, EPCs, and CAFs were mainly enriched in RNA processing, chromosome organization, and intracellular transport, indicating that stemness‐related cells were related to the development of CRC. Activated CAFs were reported to enhance the stemness of CRC cells.^24^ Researchers have found that 5‐fluorouracil combined with WNT inhibitors inhibited the activation and enrichment of CSCs and reduced the regrowth of CRC cells in the treatment of CRC.^25^ Moreover, in respect of CRC therapy, knockdown of OCT4B1 suppressed the growth of CSCs^26^; down‐regulation of miR‐144 significantly inhibited the proliferation and promoted the apoptosis of CSCs.^27^ Moreover, it has been reported that CSCs were inhibited to improve the chemosensitivity of CRC.^28^

Pseudotime trajectory analysis confirmed that the cell arrangement on the pseudotime line was CSCs and EPCs to CAFs, and 2111 genes were defined as state 2‐specific genes. There were 41 common genes obtained by taking intersection of the up‐regulated genes with state 2‐specific genes and the marker genes of CSCs, EPCs and CAFs. GO, and KEGG enrichment analysis of the 41 common genes revealed that CRC stemness‐related DEGs were mainly enriched in the positive regulation of epithelial‐mesenchymal transition, IL‐17 signaling pathway, and hippo signaling pathway. Stemness‐related genes of CSCs were once reported to be associated with the recurrence, metastasis, treatment resistance, and poor prognosis of cancers.^19^ The phenotype of CSCs induced by Hippo/YAP signal is associated with CRC recurrence.^29^

According to the univariate COX regression analysis results, fiv stemness‐related prognostic genes (TIMP1, PGF, FSTL3, SNAI1, and FOXC1) were identified. Through Kaplan–Meier curve analysis, it was indicated that the higher the expression of stemness‐related prognostic genes, the lower the survival rate of patients. Also, these stemness‐related prognostic genes have been demonstrated to play roles in the pathways of cancers. TIMP1 is critical in the tumorigenesis and metastasis of CRC and can be applied as a potential prognostic indicator for CRC.^30^ TIMP1 is also an independent diagnostic marker for CRC, and TIMP1 in the platelets promotes CRC development.^31^ PGF was previously reported to be beneficial to the treatment of breast cancer.^32^ PGF produced by CAFs facilitates neoangiogenesis in hepatocellular carcinoma.^33^ FSTL3 is a prognostic biomarker for gastric cancer (GC) and participates in the progression of GC by promoting epithelial‐mesenchymal transition.^34^ FSTL3 is also associated with lymph node metastasis, and applied as a biomarker for CRC extracellular matrix remodeling.^35^ SNAI1 is regarded as a prognostic biomarker for GC, and its high expression is closely related to the poor overall survival of the tumor.^36^ Besides, SNAI1 induces the invasion, and metastasis of ovarian cancer, and is applied for the prediction and treatment of this cancer.^37^

As confirmed by in vitro cell experiment, the expression of TIMP1, PGF, and SNAI1 was consistent with that revealed by bioinformatics analysis. Based on the findings of this study, TIMP1, PGF, and SNAI1 were identified as stemness‐related prognostic genes for the first time in CRC. These stemness‐related prognostic genes can be applied as biomarkers for the prognosis and selection of treatment strategies for CRC patients.

## 5 CONCLUSION

In this study, three stemness‐related prognostic genes (TIMP1, PGF, and SNAI1) of CRC were identified through bioinformatics analysis and in vitro cell experiment. These stemness‐related prognostic genes may provide new treatment strategies for CRC patients. However, we will continuously conduct further research in the future, because there is a lack of verification based on relevant clinical data, and no specific molecular mechanism was studied in current research.

## AUTHOR CONTRIBUTIONS

**Yan Shen:** Conceptualization (lead); formal analysis (lead); methodology (lead); writing – original draft (lead). **Siyi Ni:** Data curation (lead); formal analysis (equal); methodology (equal); validation (lead). **Si Li:** Data curation (supporting); investigation (lead); software (lead); validation (equal); visualization (equal); writing – review and editing (supporting). **Bin Lv:** Project administration (lead); resources (lead); software (supporting); supervision (lead); visualization (supporting); writing – review and editing (lead).

## FUNDING INFORMATION

This work was supported by Natural Science Foundation of Zhejiang Province (no. LY21H270007).

## CONFLICT OF INTEREST STATEMENT

The authors report there are no competing interests to declare.

## Supporting information


Figure S1.

Table S1.

Table S2.

## Data Availability

The datasets used and/or analyzed during the current study are available from the corresponding author on reasonable request.
