# Supplementary figures and images for: Role of stemness‐related genes TIMP1, PGF, and SNAI1 in the prognosis of colorectal cancer through single‐cell RNA‐seq
Source: Cancer Med. 2023 Apr 5;12(10):11611–23. doi: 10.1002/cam4.5833 (PMC10242850; doi:10.1002/cam4.5833)

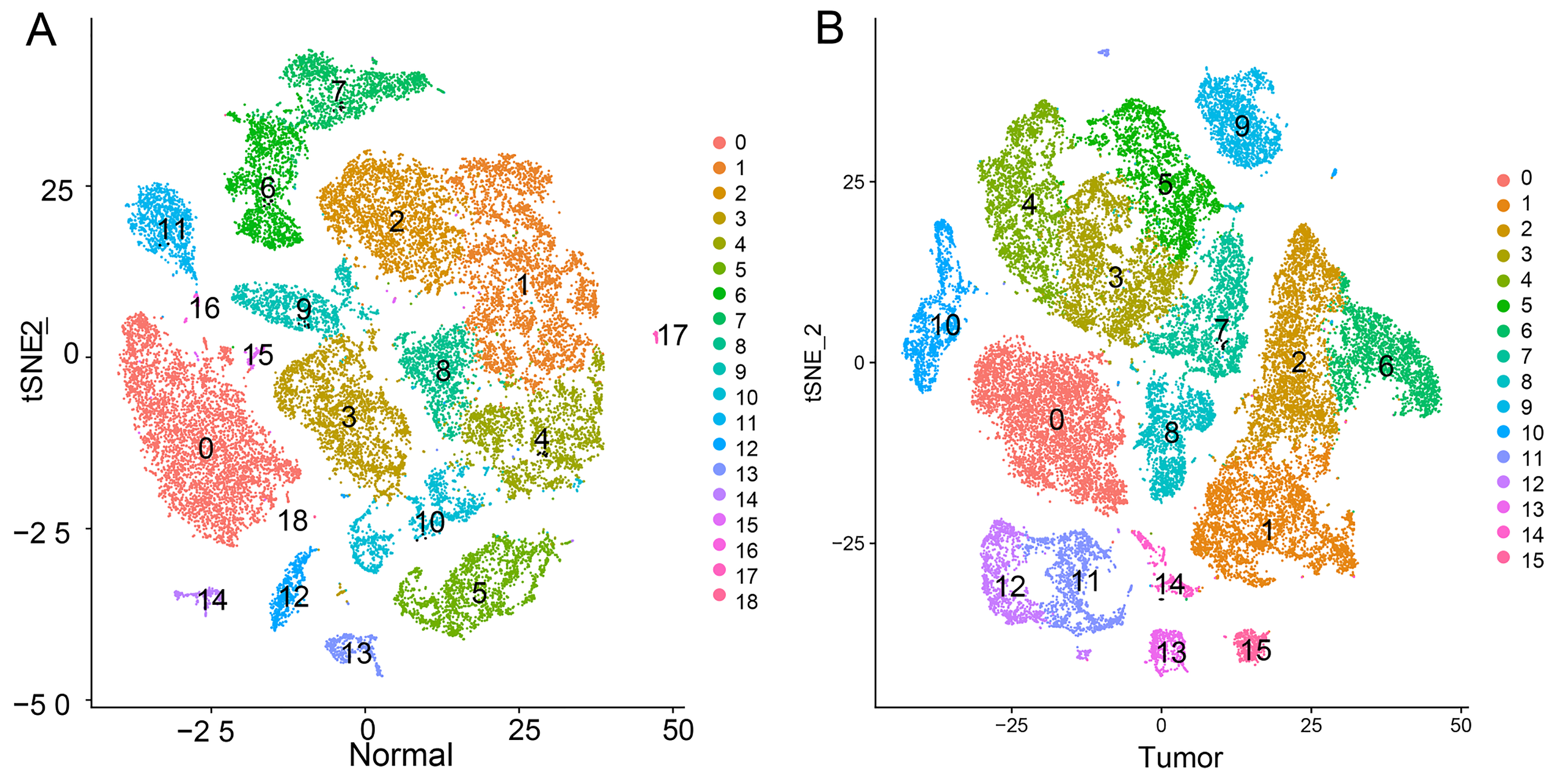

Supplement: Supplementary file 1 — Figure S1. [file CAM4-12-11611-s002.jpg]
