# Supplementary material for: Role of stemness‐related genes TIMP1, PGF, and SNAI1 in the prognosis of colorectal cancer through single‐cell RNA‐seq
Source: Cancer Med. 2023 Apr 5;12(10):11611–23. doi: 10.1002/cam4.5833 (PMC10242850; doi:10.1002/cam4.5833)
Supplement: Supplementary file 2 — Table S1. [file CAM4-12-11611-s001.doc]

**Supplementary Table 1 Cell marker genes of different cell types**

| **Cell types** | **Cell marker genes** |
| --- | --- |
| Epithelial cell | 'LCN2','ID1','KRT8','EPCAM','KRT19' |
| B cell | 'MZB1','JCHAIN','DERL3', 'CD79A' |
| T cell | 'TRBC2','CD3E','CD8A','CD4' |
| Macrophages | 'MPEG1','SAMHD1','S100A9' |
| Dendritic cell | 'CD74','CSF1R','C1QA' |
| Mast cell | 'TPSAB1','CPA3' |
| Cancer stem cell | 'PROM1','LGR5','ERBB3','CD24',  'ALCAM','ALDH1A1','ITGB1','EPCAM','CD44','MYC' |
| Mesenchymal cell | 'TSC2','CHD7' |
| Cancer-associated fibroblast | 'CXCL14','CALD1','IGFBP7' |
| Endothelial Cells | 'PECAM1' |
| NK Cell | 'NKG7','GNLY' |
| Immune Cells | 'PTPRC' |
